# Collectanea

**Published:** 1830-09

**Authors:** 


### COLLECTANEA.

**Floriferis ut apes in saltibus omnia libant,**
**Omnia nos, itidem, depascimur aurea dicta.**

### PATHOLOGY.

*Case of Enteritis, in which the extremity of the Ileum was found strangulated by the Appendices Epiploicæ.* (From Dr. O'BRIEN's *Report of the Fever Hospital, Dublin.*)

MARGARET LYONS, æt. thirty. Admitted May 31, 1829; third day of illness.

June 1. Complains of excruciating pain of abdomen, which is increased by the slightest pressure; abdomen is rather tympanitic; pulse quick, weak and irregular; countenance anxious; bowels obstinately constipated; tongue loaded.—V. S. ad unc. xx. Calomel et opium, et enemata purgantia. Twenty leeches to be applied to abdomen in the evening, with a warm bath.

2d. No improvement; had twenty grs. of calomel, and several purgative enemata, without effect; pulse weak.—V. S. and unc. xvi. Calom. et opium. Twenty-four leeches to the abdomen in the evening.

3d. No amendment; she vomits frequently; had thirty grs. of calomel since yesterday, without any observable effect on gums or otherwise.—Hirudin. xxiv. abdomin. statim. et vesp. empl. vesic. amplum.

4th. Not better; blister gave her much pain; she is now delirious, and vomits incessantly.

Died on the morning of the 5th.

I felt so intense an interest in this case, that I was determined to omit no exertion to procure an examination of the body. I accordingly sought out the patient's residence, explained to her friends the nature of her case, and earnestly requested they would permit me to examine the body, after its removal from the hospital. The request was complied with, and the dissection performed by my friend, Surgeon Trant, in my presence.

On opening the abdomen, we were soon directed to the seat of the disease by a remarkable blackness, which presented itself in the lower portion of the ileum, where it is connected with the cœcum. On examining this portion of the intestine accurately, we discovered that it was strangulated in an extraordinary manner, by a membraneous band or chord, which embraced it tightly, and completely prevented the passage of feculent matter. It was at first imagined that the stricture was effected by a convolution of the appendix vermiformis; but, on further examination, it was ascertained that the stricture was formed by two of the appendices epiploicæ attached to the cœcum and colon, which descended over the extremity of the ileum in a singular manner, and enveloped it in a loop, which completely annihilated its cavity. The whole of the peritoneal coat was in a state of inflammation; and the omentum, which was red and vascular, adhered to the intestines in several points.

Thus I had the satisfaction of ascertaining that no remedies which our art can furnish could have been of any avail in this case.

---

*Ramollissement of the Spinal Cord.* (From Dr. ABERCROMBIE, *on Diseases of the Brain and Spinal Cord.*)

This remarkable affection will be illustrated by the following important cases. In the first it was complicated with extensive inflammation of the membranes; the second shows the disease confined to the body of the cord; and the third is chiefly remarkable from its resemblance in the symptoms to an affection of the brain.

Case I. A gentleman, aged eighteen, of an unhealthy constitution, had suffered for several years from ulcers in various parts of his body, accompanied by exfoliation of bone, especially from the leg, thigh, and sacrum. For several months before the fatal attack, he had a sore on his head with caries of the bone beneath, to the extent of a shilling or upwards But he was in good general health, and was pursuing his studies at the University of Edinburgh, when, on the 24th of September, 1823, he consulted my friend Dr. Hunter, on account of sore throat with slight fever, which passed off in two days; and, on the three following days, he was going about in his usual health. On the 30th, he was again confined to the house, and complained of pain in his loins, without fever. On the 2d of October, this pain had increased; it was chiefly seated among the lower dorsal vertebræ, and extended downwards in the course of the ureters, with frequent desire to pass urine. On the 3d, the urinary symptoms were gone, the pain was diminished in violence, and it was lower down, being now chiefly referred to the sacrum. On the 4th, he continued in the same state; he was entirely free from fever; the pain in the back was by no means severe, but, as it was not removed, a blister was applied to it.

5th. The pain of the back was removed, but he complained of pain of the belly, especially about the pubis; there was some dysuria, and a feeling of numbness on the inner side of both thighs. At night there was retention of urine, requiring the catheter.

6th. The numbness of the thighs was increased, with acute darting pains occasionally shooting along them, and complete retention of urine.

7th. Perfect paraplegia of both thighs and legs, without loss of feeling; retention of urine, and involuntary stools. The most judicious treatment had been employed without any benefit.

8th. I saw him for the first time along with Dr. Hunter. There was now perfect paraplegia and complete retention of urine; pulse about ninety and soft. There was some pain, which was referred to the lower part of the dorsal region; there was no other symptom. Cupping on the back was employed, followed by another large blister, &c.

9th, 10th, and 11th. There was no change, except that the pulse wss becoming a little frequent. His mind was entire. Some pain of the back was at times mentioned, but it was not severe, and he made no other complaint. Perfect palsy of the limbs continued, and the numbness was extending upwards upon the abdomen.

12th. The numbness was extending upon the thorax: there was very little complaint of the back, but acute darting pains were complained of extending along both arms. The sore on the head being examined, and the opening enlarged, the bone was found carious, and some matter was discharged from it by a very small opening. In consequence of this appearance, a perforation was made by a small head of a trephine, but no disease was found beneath the bone. In the evening, his pulse having become more frequent, further bleeding was employed with relief.

13th. No change; his mind was quite entire, and he made no complaint: stools involuntary.

14th. The urine came off without the catheter, on raising him up into an erect posture. Pulse frequent and feeble; strength sinking. He died in the night, having continued quite sensible until about six hours before his death. There never had been the least attempt at motion of the lower extremities, but the sensibility remained.

*Inspection.* In the brain all was quite sound, except some old thickening of the dura mater in the neighbourhood of the diseased bone. The bone was carious and very thin to the extent of a half-crown piece : around this portion it was thickened, especially on one side, where it was fully double the natural thickness. There was no effusion in the head, and no appearance of any recent disease.

In opening the spinal canal, some purulent matter flowed out during the sawing, from about the middle of the dorsal region ; and one of the vertebræ at that place was found considerably carious. The canal being opened, there was found most extensive deposition of flocculent matter, of a purulent appearance, on the outside of the membranes of the cord : it was most abundant for some inches about the lower part of the dorsal region, but likewise extended upwards to the fourth cervical vertebra. The dura mater of the cord being laid open, bloody sanious fluid was discharged from beneath it; and the pia mater was found highly vascular. The substance of the cord was found most extensively destroyed in its structure along nearly the whole extent of the dorsal portion. The anterior columns of this part were completely disorganized and broken down into a soft diffluent pulp; on the posterior part, the cord was more entire. When the whole cord was taken out and suspended, it hung together by the posterior columns of the dorsal portion, while the anterior part of it fell off entirely in a soft diffluent state. The parts above and below the diseased portion were quite firm and healthy.

Case II. A man, aged fifty-six, in the last week of March 1823, was much exposed to cold in travelling on the outside of a coach : after which, he was seized with pain of the right arm and leg, most severe about the shoulder, but affecting the whole side; and there was considerable headach. He soon perceived some loss of power of the affected limbs, which began at the upper part of the arm, and extended downwards so gradually that he was able to write distinctly, after he had lost the power of raising the arm or bending the elbow. The leg then became affected in the same gradual manner, and, after about ten or twelve days from the commencement of the disease, the whole leg and arm had become completely paralytic. Some pain continued in the parts, and was sometimes severe, especially in the leg. About this time he was first seen by Dr. Moncrieff, who found the pulse ninety-six, and rather sharp. He was quite sensible, and still complained of some headach, and of pain extending from the shoulder along the affected arm and leg. Repeated bloodletting, blistering, purgatives, &c. were employed, and the headach was removed. The other symptoms continued as before; the right leg and arm were completely paralytic, and sometimes very painful; pulse eighty-four, and rather weak; his mind quite entire. He continued in this state till about the 26th of April, when the left arm became paralytic rather suddenly: it did not, however, become so completely motionless as the limbs of the right side, and the left leg was not at all affected. The pulse was now feeble, and his general appearance expressive of exhaustion. I saw him first about this time. There was slight delirium, which however passed off again; and he continued quite sensible, and even cheerful, without any pain, except occasionally in the right leg, till the 7th of May, when he became again delirious ; the pulse 120, and weak. On the 8th, he lay in a state of stupor, muttering incoherently, but answering questions distinctly when he was roused. He died on the morning of the

9th, having lost his speech a few hours before death. For the last eight or ten days there had been extensive gangrene on the sacrum.

*Inspection.* Every part of the brain was found in the most healthy state. Much bloody fluid was discharged from the spinal canal into the cavity of the cranium before the spine was laid open. On laying open the spinal canal, the cord was found in a state of complete ramollissement, from the second to the last cervical vertebra; the parts above and below were quite healthy.

The following case shows the disease running its course with much greater rapidity, and with a different train of symptoms.

Case III. A boy, aged seven, had been indisposed from the 18th to the 20th of May, 1823, but so as to attract little notice. There had been some headach and slight feverishness, for which he took purgative medicine, and on the morning of the 22d he seemed almost well. About two o'clock in the afternoon of that day, he was seized with severe and general convulsions. I saw him soon after this, and found him confused, incoherent, and partially comatose; the pulse sixty and weak; face pale; the bowels were slow, and some worms had been passed. The usual remedies were employed with little effect.

23d. In the morning he was partially comatose, the eye fixed and insensible. In the course of the day he became less comatose, but incoherent, with much talking and screaming; complained of headach, and was impatient of light. In the evening there was slight appearance of squinting, and in the night some convulsions; pulse very variable, being sometimes rapid and sometimes slow; the bowels were very obstinate, but yielded to repeated doses of croton oil.

24th. Seemed much better: eye natural, face pale, pulse 120, bowels kept open by the croton oil. He was quite sensible, and said there was still some headach, but did not appear to suffer. He continued in this favorable state until early in the morning of the 25th, when the convulsions returned with much severity, after which he sunk into a low oppressed state, and died early in the afternoon.

*Inspection.* There was no effusion, and no appearance of disease in the brain. On removing the brain, a considerable quantity of fluid flowed from the spinal canal; and, on laying open the canal, there was still a good deal found between the cord and the external membrane. The cord was healthy at the cervical portion, but in the upper part of the dorsal region it was remarkably softened and broken down. This appearance extended for several inches, but varied in degree. At one place a complete separation took place in attempting to raise the cord, the part falling down into a soft diffluent pulp through its whole diameter. From the middle of the dorsal portion it was quite firm and healthy. The inner membrane of the cord was dark coloured, highly vascular, and showed evident marks of inflammation at the part corresponding with the softened portion of the cord.

---

*Cases of Hematosis.* By Dr. Samuel Jackson. (*American Journal of Medical Sciences.*)

A deficiency or excess of blood, it is well known, may exist, constituting Anemia, and Hyperemia or plethora, and be productive each of peculiar phenomena. But a deficiency or excess of some one of the different elements of the blood may also prevail, and be the cause of particular states of the economy, or generate special symptoms, amounting to a pathological condition. In the highly marked lymphatic temperament, the watery and albuminous principles predominate over the fibrinous or globular and colouring elements, and modify, in consequence, the phenomena of disease. The highly sanguine temperament presents the opposite extreme, and fibrin or globules, and the colouring principles, are in excess, equally impressing a peculiarity on morbid phenomena. The circumstance, however, to which I now wish to attract attention, is the sudden diminution, amounting almost to a disappearance, of the hematosine or colouring principle of the blood.

The nutritive sanguine humor is formed from the nutritive principles furnished by the aliment, which are concocted into blood by the various processes through which they pass. In what manner hematosine, or the colouring principle, is formed, what organ is charged with its production, or what is the process creating it, we have no knowledge. Respiration appears to have some agency in its formation, yet this function may rather be presumed to perfect the process of its production, than to form it exclusively; for in the instances in which this principle was deficient, which have met my observation, respiration was wholly unimpaired.

Case I. Miss M'C———, aged nineteen, lymphatic temperament, hair of light brown, eyes a light grey, complexion fair, full embonpoint, skin soft and of delicate texture, limbs round and smooth, chest expanded. She has enjoyed uniformly good health; menstrual function commenced without difficulty, and has continued until the present period, uninterruptedly, though always very slight.

After unusual exertion, she noticed that slight exercise produced lassitude and fatigue, excited palpitations of the heart, a throbbing, obvious to the eye, above the sternum, and on the right side of the neck, and caused a sense of beating in the head, and through the limbs to the ends of her fingers. These symptoms increased, and the pulsation above the sternum and on the side of the neck, became permanent, although feeble when she was sitting. The augmentation of the symptoms finally arrived to such a degree as to prevent all active exertion.

I visited her on January 4th, 1830. As she had been perfectly quiet, nothing could be observed but a sallowness of complexion, and a slight throbbing above the sternum and right clavicle. I explored the chest with the stethoscope. The respiration was clear and distinct in every region of the thorax; indicating, as might have been expected from her finely expanded chest, an admirable pair of lungs. The rythm of the heart was natural; the impulse rather strong, and sound somewhat louder than usual, but not to an extent that could be regarded as morbid, or belonging to organic change. No purring sound, throbbing, or uncommon noise, under the right clavicle or superior end of the sternum, (marks of aneurism,) could be heard. The appetite was excellent, diet too full, digestion perfect; bowels perfectly regular, and evacuations natural. The menstrual discharge, which had declined rapidly since the invasion of the present symptoms, had not returned at the last period, the first of the month. The tongue was clean and moist, but was without colour, as were the lips, gums, and inside of the mouth; the pavilion of the ear, when held to the light, was nearly diaphanous. When quiet, she experienced not the slightest inconvenience, and would not, she remarked to me, be conscious of any thing unusual in her situation.

I now requested her to ascend a flight of stairs, and return. On entering the room, she threw herself into a chair, with an exclamation that "she was nearly gone." She exhibited a picture of exhaustion: her limbs hung relaxed, her head fell on her shoulder, she panted violently for breath; the throbbing above the sternum and on the sides of the neck was violent, but strongest on the right; the heart palpitated excessively; its contractions were tumultuous, so as not to be counted, and were sharp and strong. This state continued about five minutes, when it began to decline, and gradually terminated. I examined with great care the right clavicular region, to discover the existence of a tumor, and, though the pulsation was very distinct to the feel, yet nothing like a tumor could be detected.

From a review of the case, I could not arrive at a satisfactory conclusion. I was strongly disposed to regard it as an aneurism or dilatation of the aorta, at its arch, or of the innominata. It resembled in many respects the cases of aneurism recorded by Mr. WARDROP, especially his fourth case: yet it exhibited many features of anemia; but, as no losses of blood had been sustained, the symptoms could not well be attributed to that cause. In this uncertainty, I limited my directions to a reduction in some degree of the diet, to take a mild cathartic, and to keep perfectly quiet.

The symptoms continued without abatement, and, having a consultation with a friend who is engaged in surgical practice, I invited him to examine this case. After an attentive observation of the phenomena it presented, he was decidedly of opinion that an aneurism of the aorta or innominata was the cause producing them. On the 10th of January, acting on the impression of the existence of an aneurism, I directed V.S. to ten ounces, with an abstemious diet.

The next day, 11th, all the symptoms were greatly aggravated. She was now scarcely able to leave her chair; every attempt to walk about the room excited panting and palpitations; the throbbing in the neck was more violent, and it was with difficulty she was got up stairs to bed. The blood drawn consisted almost entirely of serum, the crassamentum being very small. To relieve the urgency of the symptoms, warm wine whey, with a calming potion, were ordered. I had no longer a doubt, from the result of the bleeding, that the case was one of anemia, or what is nearly of the same nature, a deficiency of the fibrin and hematosine of the blood. The following powder was prescribed: Phosphas Ferri, gr. v.; Sulph Quinæ, gr. ss. every four hours; the diet was made more generous.

January 14th. Symptoms diminishing; palpitations lessened. Powders continued.

21st. Throbbing in the neck and above the sternum has diminished very considerably; she can now ascend and descend the stairs without the distress and faintness formerly experienced, though palpitations continue to be induced. Continue powders.

28th. Progressive amendment; menstrual discharge returned; colour returning to the cheeks, lips, and tongue. Continue treatment.

February 10th. Symptoms have entirely disappeared; complexion restored to its usual healthy aspect. Was last night at a dance, and experienced no inconvenience from the exertion. Omit the treatment.

March 10th. Health continues perfect; exercises daily, and suffers from none of the former symptoms.

*Remarks.* The symptoms of the above case would probably be accounted for by many as nervous. But this term, unless it be defined, and a positive idea attached to it, is no explanation of the phenomena, and can give no clue to a plan of treatment. It is not probable that they had their origin in the nervous system, for the mind and feelings were perfectly tranquil. The subject of the case is a girl of excellent sense, has never shown any tendency to a morbid sensitiveness of nerves, and moral impressions produced but slight effect in exciting the symptoms. Besides, we do not know that nervous affections, as they are termed, can occasion the disappearance of the hematosine or colouring principle of the blood.

Taking a review of the whole of the preceding case, it appears to me evidently to have depended on a morbid alteration in the proportion of the constituent elements of the blood, the fibrin and hematosine or colouring principle being deficient.

### PRACTICAL MEDICINE.

*On the Employment of Metallic Mercury in Cases of Volvulus.* By M. Ebers. (*Journ. der Pract. Heilk.*)

The author gives a rapid sketch of the various means that have been recommended in intestinal intus-susception, and then enters more at length into an account of the employment of quicksilver in such cases. In the use of this remedy there is much less danger than has been supposed by many physicians; but it is doubtful whether it is equally applicable when the superior portion of intestine has slipped into the inferior, as when the reverse has happened. It would appear that it can only be of use when a lower portion of intestine is invaginated in an upper: but as it is absolutely impossible to distinguish this difference during life, we must take the chance, when symptoms occur of such a nature as to demand the treatment referred to.

Two cases are related. In the first, a labourer of advanced age, who, after a very active life, had become comparatively indolent, was subjected to frequent attacks of pain in the belly, with constipation. Various remedies were prescribed; but at last, probably from a severe fit of indigestion. the pains increased very much in severity, and were not relieved by any treatment. Frequent vomiting came on, and at length fecal matter was discharged by the mouth. Every symptom announced the speedy dissolution of the patient, and, as a last resource, it was determined to give quicksilver. One ounce was given, and immediately after a cup of broth. Two hours after, two ounces were administered. The vomiting now ceased, and the patient slept calmly. When he awoke he still complained of pain, but it was much diminished. He was persuaded, with some difficulty, to take a third dose of the remedy. In about an hour after this last dose, a loud borborygmus was heard in the belly; the patient was extremely restless, screamed out violently, and it was believed he would quickly die: but suddenly he rose from his bed, and discharged from the anus an immense quantity of fecal matter, and in the course of a very short time he had several stools. The mercury was seen divided into small globules in the evacuations. The cure was now completely effected.

In the second case, a woman had suffered violent colic for many days, in consequence of obstinate constipation. She vomited incessantly, and discharged fecal matter by the mouth, together with a brown chocolate-coloured mass. Her face exhibited great anxiety; skin cold, pulse small and rapid, great debility. The danger was imminent. Great tenderness over the whole surface of the abdomen, which had a doughy feel, from the immense accumulation of feces. The patient was in a constant state of agitation, and uttered the most piercing cries. A warm bath and clysters were tried, without benefit. Every medicine was immediately rejected, with violent vomiting. M. Ebers now determined to give four ounces of quicksilver at a dose. In a few minutes after having taken it, she became more tranquil, although the pain continued with equal severity. In an hour, two ounces more of the metal were given, and the patient soon fell asleep. She had not dozed more than half an hour, when she awoke in an apparently dying state; but at the same moment she had a very copious and fetid evacuation, which was followed by several more. In each evacuation mercury was detected. After this free action upon the bowels, the patient was completely relieved, and in a few days she was restored to her accustomed health.

It will be observed, that in both these cases the mercury acted in a similar manner. At first there was a state of comparative calmness after taking the remedy, and then sleep. When the patients awoke, the symptoms were very alarming for a short time, but copious stools followed, and health was quickly restored.—*Bulletin des Sc. Medicales.*

---

*Acupuncturation in Rheumatism.* The following interesting cases, showing the striking effects of acupuncturation in the removal of rheumatic pains, are related by Dr. Jos. BERNSTEIN of Warsaw, in Hufeland and Osann's Journal der Practischen Heilk., August 1828.

CASE I. N., a man, 54 years old, of a sound constitution, from exposure to cold during the winter, became affected with a violent rheumatic pain of the upper part of the arm. A variety of remedies were employed without effecting any permanent removal of the disease. Six months had elapsed since the commencement of the attack, when Dr. B. was called in. The affected shoulder was neither red nor swollen, the patient could move the arm easily forward and without pain ; but by the motion of the arm either upwards or backwards the pain was greatly increased. The pain was not augmented by pressure. The health of the patient was otherwise tolerably good. A large blister was directed to the arm in the neighbourhood of the deltoid muscle, at which place the pain was the most severe during the motions of the arm ; the blister was kept open for four weeks, at the termination of which period an evident diminution of the disease was experienced : circumstances relating to the patient's occupation now rendered it necessary that the blistered surface should be allowed to heal, although the disease was not yet entirely removed. The patient continued six weeks without medicine of any kind : the motions of the arm upwards and backwards were still painful. Acupuncturation was then resorted to. On the first day, five needles were inserted near the insertion of the deltoids : three, 11-2 inches, in a perpendicular direction, and two, after being passed to the same depth perpendicularly, were then directed obliquely in the course of the muscles of the arm. After 15 minutes the needles were removed in the same direction in which they were inserted. The introduction of the needles caused little pain, but their extraction somewhat more. The patient was now directed to move his arm ; and he was of opinion that the disease was already evidently diminished. On the second day, less pain was felt at the spot where the needles were inserted, but still some was experienced when he attempted to raise his arm upwards. Five needles were again inserted and left in twenty minutes. The pain was evidently diminished. Third day, the pain was felt lower down, and the needles were here inserted; pain diminished. In this manner constantly for two weeks was the operation repeated on different parts of the arm at which the pain was experienced, and finally the disease was entirely removed. " Seven months have now elapsed," says Dr. B., " and notwithstanding this individual has been exposed to cold and dampness, there has been no return of the pain."

CASE II. A. L., twenty-seven years old, of a sound, robust constitution, contracted, by exposure to cold, a rheumatism of the right lower extremity, extending from the hip-joint and glutei muscles to the calf of the leg. Its removal had been attempted by diaphoretics internally, and frictions with the volatile liniment to the seat of the disease : by this treatment the pains were increased rather than diminished. Dr. B. saw the patient about six weeks from the commencement of the attack : the pulse was full, not accelerated, but very strong ; venesection to 12 oz. was directed, to be followed by 20 leeches to the affected parts. By this the pain in the thigh and neighbourhood of the hip-joint was very considerably diminished, but not entirely removed: the patient also complained of a weakness of the foot, preventing him from treading upon it with firmness. He was now placed upon a proper diet and regimen, with no other medicine than was necessary to preserve an open condition of his bowels: after several days a pain was experienced extending more into the hollow hip; the patient could not walk without pain; he could, however, tread upon the sole of the foot, and stood firmly on both legs, but could not turn the foot on the affected side, in all directions, without some pain. No flattening of the buttock on the side diseased. A blister was directed, but objected to by the patient; acupuncturation was then proposed, aud agreed to: ten needles were inserted, near the hip-joint, in a perpendicular direction, and allowed to remain in fifteen minutes: immediately upon their extraction, a diminution of the pain of the hip was experienced. On the next day, pain of the hip less, but it was more severe in the buttock: here ten needles were introduced, and five at the hip-joint. Third day, hip free from pain, and considerably less pain was also felt at the buttock: for twelve days a dozen needles were daily inserted at different parts of the thigh and leg; and by degrees the pain was entirely removed. "Ten months have now elapsed, and the patient has had no return of the disease. It is proper to remark, that, during the whole time acupuncturation was employed, the patient followed his usual occupations."

Case III. W. S., twenty-five years of age, had been for twelve months occasionally affected with a pain of the head, particularly of the left temple. The pain continued usually for a day or two, and was often so severe as to prevent the patient from attending to his business. Subsequently the pain recurred regularly every month; it was preceded by no chill, nor attended by any fever or perspiration: the urine was, however, clouded. The case was at first treated for a Febris intermittens larvata: the patient happening, however, to witness the operation of acupuncturation, requested that it might be tried in his case; he was accordingly directed to remain without medicine until the next paroxysm occurred, when a fold of the skin being raised at the seat of the pain, through it were inserted six steel needles; after fifteen minutes they were removed, and it was found that the pain had entirely disappeared. The ensuing month the pain again returned, but with less severity; the introduction of the needles was repeated, and in ten minutes the pain was entirely gone, nor has it again returned, although eight months have elapsed since the operation was last performed: previously, for a twelvemonth, the pain recurred regularly every month.

Case IV. This was an inflammatory affection of the left arm, extending from the shoulder to the elbow, and produced by the patient having, three days before, carried a basket heavily loaded with wood up a steep hill. By the advice of a surgeon, she had lost three cups of blood, had twenty leeches applied to the affected arm, followed by frictions with the volatile liniment. When Dr. B. saw the patient, the arm was greatly swollen and red; the slightest touch, and every motion of the limb, increased the pain; the pulse quick, full, and hard. The patient was upwards of forty-five years old, of a robust constitution, and had previously enjoyed good health. Notwithstanding the symptoms of this case appeared to call for the continuance of depletory measures, yet several circumstances induced Dr. B. previously to try the effects of acupuncturation: fifteen needles were accordingly inserted at the seat of the disease, in various directions; when, after fifteen minutes, they were withdrawn, the patient declared that her sufferings were much lessened, and she was able to move the arm freely. She besought Dr. B. to permit her to return the following day, in order that, by a repetition of the operation, her pain might be entirely removed. Nothing more, however, was seen of this patient for two months, when she came to the doctor's house in company with another patient. She then stated that the day after the operation, all pain having ceased, and the free motion of the arm in every direction having returned, she had therefore not thought it necessary to trouble the doctor. No remedy of any kind, excepting the acupuncturation, had been employed.

Case V. K., a man about forty years old, had for two years laboured under a pain of the loins, which finally arose to such a height as to prevent him from following his business. Every motion of the lower extremities augmented the pain, and a recumbent posture was the only one in which any ease could be obtained. There was no fever, the appetite was diminished, the tongue covered with a white fur; great tendency to constipation. An interrupted state of the circulation through the abdominal vessels was evinced by the sallow complexion, uneasy feelings in the abdomen, frequent griping pains in the region of the stomach, and the occasional fits of peevishness to which the patient was subject, without any external cause; no hardness or swelling in the abdomen could be detected. The patient was, during three weeks, treated by gentle resolvents, and an occasional cathartic. Under these remedies his general health improved; tongue cleaner, appetite restored, and the inclination to peevishness removed; but the pain in the loins was in no degree abated. For two weeks all remedies were discontinued, at the end of which time, the pain still continuing, five needles were inserted on each side of the loins; after fifteen minutes they were withdrawn, and the pain was found lessened at the place of their insertion, but removed lower down. For ten days the operation was repeated daily on that part to which the pain was successively removed, and the patient was then enabled, without inconvenience, to bend and turn himself in every direction, which he had been unable to do for two years. "Twelve months have now elapsed since the last performance of the operation, and the condition of the patient is still the same."

Case VI. A man, about forty years old, had for eight weeks been affected with a chronic rheumatic inflammation of the left eye, with opacity of the cornea. The usual remedies had been employed without effect. When Dr. B. saw the patient, he still complained of pain in the head and eye. Blisters were directed to the back of the neck, to the fontanels, and to both arms, with various remedies internally; these produced no effect. For two weeks all medicines were suspended: acupuncturation was now performed, and the inflammation of the eye and pain of the head were entirely removed; the opacity of the cornea was also considerably diminished. The operation was repeated daily for two months, to ascertain its effects upon the latter, when, to the astonishment of the doctor, he found that the spot upon the cornea was reduced to less than one half its original size, and its opacity so much diminished that it interfered very little with the patient's sight.

---

*Cases of Amenorrhœa treated with Strychnia.* (Bardsley's *Hospital Facts, &c.*) As amenorrhœa is a frequent source of several of the most troublesome affections to which females are liable, it is of importance to be able to name any new remedy which will be found occasionally serviceable in promoting a regular and natural flow of the catamenia. Previously to my experiments with the strychnia in this disease, I had been in the habit of employing aloes more extensively than any other medicine; and I can add my testimony to that of the venerable Dr. Hamilton, in favor of the utility of purgatives in exciting the action of the uterine vessels. In many cases, I have succeeded in obtaining a renewal of the menstrual discharge, by a steady but active administration of the aloetic pill alone.

It is foreign to my purpose to enter upon an inquiry into the causes and consequences of amenorrhœa, as my principal object is to state facts; I shall therefore proceed to illustrate the successful administration of strychnia in my hands, in some instances of suppressed menstruation.

Case I. Phœbe Lowe, thirty-three years of age, unmarried, admitted an out-patient, June 4th, 1825.

She stated that she had not menstruated during the last five months. She attributed the obstruction of the menses to cold. She was languid and weak. Her appetite was impaired, countenance pallid, and strength diminished. Bowels rather costive; pulse eighty-two; tongue white. Having brought the bowels into a natural state, by the exhibition of purgatives, I commenced with the sixth of a grain of strychnia, three times in the day.

June 16. She has not as yet derived any benefit from the alkali. Dose to be increased to the fourth of a grain at the same intervals.

July 12th. Her general health was much improved, and her appetite is better. She has experienced rather painful sensations in the hypogastric region, during the last few days. To continue.

24th. The menses have appeared in small quantity since the last report. The uneasiness in the hypogastrium has left her. To continue.

30th. She improves in health daily, and is anxious to follow her usual employment in the cotton factory. Her appetite is very good, countenance more lively, and strength greatly increased. I desired her to suspend the further use of the medicine, giving her a strict charge to call at the Dispensary, and to inform me if the catamenia had become regular. Agreeably to my request, she attended on the 14th of October, and said that she had menstruated twice rather copiously since she saw me. Discharged cured. This woman married shortly afterwards, and is now the mother of two children.

Case II. Ann Gilldike, aged thirty years, unmarried, admitted an out-patient, 24th February, 1825.

This woman had imprudently exposed herself to wet and cold for several hours when the menses were last present, and they have not since returned. Her complaint is of six months' duration. Countenance pale, body emaciated, appetite impaired, bowels natural. She stated that she had been under medical treatment for more than four months, but had not derived any benefit from the remedies employed. I immediately commenced with the sixth of a grain of strychnia three times daily.

March 14th. No change in her symptoms. Dose of alkali to be increased to the fourth of a grain, at the same intervals.

29th. She finds herself considerably better, and, judging from her present feelings, thinks that the catamenia will shortly appear. Bowels regular, spirits cheerful. To continue.

There was a slight uterine discharge on the evening of the 21st, but it ceased early on the following morning. Her looks and general health are surprisingly improved. To take half a grain of the alkali twice daily.

May 24th. On the 18th the catamenia appeared, and continued until the 22d: their colour and quantity were perfectly natural. She makes no complaint but of debility. To persevere with the strychnia, in the dose of a fourth of a grain three times daily. She attended at the Infirmary on the 6th of July, and mentioned that the menses had occurred at the usual time, and that she felt herself as well as at any former period of her life. She was discharged cured. This patient has continued well ever since.

---

### SURGERY.

*Treatment of Inverted Nails.* (*Révue Medicale.*) Cases of this kind appear trifling, and are sometimes very unwarrantably neglected, both by the patient himself and the surgeon, until great suffering arises. Various modes of practice have been adopted; amongst others, evulsion of the nail, which necessarily gives great torture to the patient, and has been known to destroy him. The following plan is employed with success by Dr. Biessy, of Lyons. He scrapes the healthy portion of the nail to an extremely thin layer, and then repeatedly touches it with lunar caustic, until the anterior portion shrivels, and separates spontaneously from the flesh beneath. Small bits of lint are then inserted between the nail and the flesh, until the new nail has become sufficiently large, which is usually the case in twenty or thirty days after the operation.

---

*Imperforation of the Cervix Uteri: Operation successful.* Delpech relates this case. A young woman, ætat. twenty-two, who had never menstruated, was attacked at the age of seventeen with colic, which gradually increased in severity. She became pale, emaciated, melancholy, and lost her appetite. Twice in the course of one year the pains in the abdomen, which always commenced in the hypogastric region, were so severe as to endanger the life of the patient; they gave rise to violent fever and delirium. Violent paroxysms of hysteria followed. M. Delpech was now consulted. In the hypogastrium, a spheroid and moveable tumor was felt, which was very tender, when touched; it impeded the motion of the patient, and rose as high as the umbilicus. It was presumed that this tumor was the uterus distended by the retained menses, but no trace of the cervix or os uteri could be detected. A puncture was made in the most depending part of the tumor in the vagina, and about one pint and a half of a brown inodorous fluid was discharged, of an oily consistence. The tumor was now diminished to half its former size. The discharge continued for several days, and the opening that had been with a trocar could be felt in the vagina. An irritable state of the breasts, which had before existed, was now completely relieved. The patient has menstruated twice since the operation, and no unpleasant symptoms had occurred. (*Révue Medicale.*)

---

*Contraction of Parts after Burns.* M. Dupuytren lays it down as a principle, that in cases where fibrous bands, resulting from unfavorable cicatrization, impede the motion of one or several articulations, the parts should be set at liberty by transverse incisions, without destroying the cicatrix itself. Surgeons, in general, adopt the mode of removing the original cicatrix, and then they endeavour, by artificial efforts, to form one more regular, which may not interfere with the action of the parts implicated in the injury. The following is an example of his practice.

A child, nine years old, was admitted into the Hôtel Dieu, with a deformity of the hand, the consequence of a burn which had happened a considerable time before. The little finger of the left hand was bent forward, and was confined to the palm by a membraniform bridle, the folds of which terminated in a sort of fibrous cord, which extended between the first and second phalanges. The ring finger was less burnt, but was also attached to the palm of the hand by a falciform bridle, which made it describe a regular segment of a circle. The connecting band extended from the tip of this finger to its metacarpal extremity, and was about one and a half inches in length. The middle finger was bent forward like a hook; its free extremity corresponding to the articulation of the first with the second phalanx. A tight and thick bridle fixed it in this situation, and prevented its extension. The object here was evidently to extend the fingers, and to retain them in that position until new and regular cicatrices had formed. M. Dupuytren made above and below each fibrous band a transverse incision, which divided the whole substance of the skin. He then extended the fingers, dressed the wounds, and applied a broad splint upon the back of the hand, ensuring the constant extension of the fingers by a bandage. The wounds cicatrised, and their edges remained apart by the formation of a new cutaneous tissue. The exuberant fleshy granulations were destroyed with lunar caustic, and at the end of a few days the splint was removed, when the new cicatrices, if not completely formed, were in such a state as to warrant their being left to the efforts of nature. The success of the case was very gratifying.—*Journal Complémentaire, &c.*

